# Predicting IVF live -birth probability using time-lapse data: Implications of including or excluding age in a day 2 embryo transfer model

**DOI:** 10.1371/journal.pone.0318480

**Published:** 2025-02-25

**Authors:** Shabana Sayed, Bjørn Molt Petersen, Marte Myhre Reigstad, Arne Schwennicke, Jon Wegner Hausken, Ritsa Storeng

**Affiliations:** 1 Klinikk Hausken, IVF and Gynaecology, Haugesund, Norway,; 2 BMP Analytics, Consultancy, Viby J, Denmark,; 3 Norwegian National Advisory Unit on Women’s Health, Oslo University Hospital, Oslo, Norway; School of Sciences and Languages, Sao Paulo State University (UNESP), BRAZIL

## Abstract

The primary objective of this study was to develop predictive models for the likelihood of live births following In Vitro Fertilisation (IVF) treatment, based on a retrospective analysis of time-lapse data from Day 2 embryo transfers at Klinikk Hausken, Norway. This analysis encompassed 1,506 IVF treatment cycles, which included 865 single and 641 double embryo transfer cycles, totalling 2,147 embryos transferred.

The model covariates included nucleation error, timing of two-cell stage (t2) and duration between t2 and the three-cell stage (t3). The predictive ability was assessed using Area Under Curve (AUC). Generalised Additive Mixed Models (GAMM) were utilised to address clustering effects from Single Embryo Transfers (SET) and Double Embryo Transfers (DETs), as well as the non-linear effects of female age and t2 timings. A stratification of age and model scores demonstrated the impact of incorporating age into the model. The” Base Model, not incorporating age, achieved an AUC of 0.641, while the “Age Model”, using maternal age, significantly enhanced AUC to 0.745, as estimated through bootstrap analysis.

However, when the Age Model was subjected to average ages across three respective age intervals, the AUC values were comparable to the Base Model, rather than the original Age Model scores.

Adjusting the Intracytoplasmic Sperm Injection (ICSI) timing by ± 2 hours, purely as a theoretical exercise, has minimal impacts on model predictions. This highlights the value of including t2 despite fertilisation timing variations between ICSI and IVF.

The Age Model did not show superiority in predicting live birth within single treatment cohorts. However, given its distinct AUC values for broader age ranges, the Age Model can serve as a counselling tool on live-birth probabilities. With further validation, we suggest only using the Age Model for general counselling, while the Base Model is preferable for the embryo selection decision support.

## Introduction

One of the key factors determining successful outcomes following In Vitro Fertilisation (IVF) treatment is the selection of embryos with the highest potential for implantation and live birth [1,2]. As single embryo transfers (SET) have become more preferred to reduce risks associated with multiple pregnancies, the importance of effective embryo selection methodologies has increased. Standard morphology evaluations have been the benchmark for embryo selection [3,4]. However, this subjective method, involving embryo assessment at specific time points, results in both inter and intra-observer variability. The variability constraints the accuracy of predicting implantation and live birth outcomes [5–7].

Introduction of time-lapse imaging (TLI) technology in IVF has provided the embryologists with a multitude of additional non-invasive embryo selection and de-selection biomarkers, both morphologic and morphokinetic [8–10], further aimed at promoting SET. Nucleation error phenotypes [11–13], time-lapse imaging derived morphokinetic variables [11,14–17] and cleavage anomalies observed by TLI [18–22] have been correlated with treatment outcomes. However, when comparing the effectiveness of TLI-based embryo selection to that of standard morphology evaluation for predicting clinical outcomes, the results remain inconclusive [23–25].

The significant heterogeneity among patient populations, day of transfer, insemination methods, culture conditions and variability in the time-lapse devices used in the study analyses may have all led to this inconclusiveness [26–30].

Time-lapse biomarkers that consistently predict clinical outcomes such as blastocyst formation, implantation and clinical pregnancy rates across clinical studies have been utilised to develop hierarchical embryo selection models [15,25,31]. These initial models were altered by incorporating varied sample sizes, statistical approaches and new selection and de-selection biomarkers. These approaches have resulted in the development of either centre-specific, multicentre, and generally applicable algorithms for embryo selection [25,31–35].

Early morphokinetics algorithms predicting blastocyst development were later validated and adapted in multicentre studies [15,25,31,36,37]. However, inconsistencies in the predictive ability of many of these models during internal and external validation may have restricted their full implementation in clinical practice [38–43]. Subsequent studies have indicated a potential improvement in clinical outcomes [22,32]. Randomised controlled trials (RCT) by Rubio et al. (2014) showed potential benefits of using TLI in embryo selection, with a significant increase in ongoing pregnancy rates in the TLI group compared to the control group [37]. However, variations in culture conditions and the use of hierarchical time-lapse embryo selection models between the groups have potentially weakened the conclusiveness of these findings. A randomised sibling study by Yang et al. (2014) showed a significant improvement in ongoing pregnancy rates for euploid embryos selected based on their morphokinetics scores. However, the use of different culture conditions for the study groups reduced the significance of their conclusions [44].

Despite the sparse concrete evidence from RCTs for a clinical benefit of TLI, continuous monitoring of embryo development in an undisturbed environment offers more information and may enhance identification of good-prognosis embryos for clinical use [45]. Several factors, including patient and treatment characteristics alter morphokinetics timings and influence embryo selection model performance, especially when implemented in diverse clinical setting with heterogenous patient populations and culture conditions [46–48]. Among various patient-related confounding factors, maternal age stands out as a critical determinant for implantation success [40,49]. Maternal age is a known factor in reduced IVF success rates, often attributed to increased oocyte aneuploidy, decreased mitochondrial function, and compromised pre-implantation embryo development [50–53]. Initial studies investigating the impact of maternal age and morphokinetics found no significant effects [54,55]. However, Liu et al. (2019) observed notably different implantation rates for morphologically similar embryos derived from different female age groups [40]. This disparity may be due to differences in chromosomal or genetic makeup of embryos from younger versus older patients [40]. Additionally, female age was found to significantly affect four morphokinetic parameters; the time to two-cell (t2), time to four-cell (t4), time to blastocyst (tB) and the time between morula and start of blastulation (tM-tSB) [56]. However, Kirkegaard et al. (2016) emphasised the concern of potentially overvaluing the statistical relevance of observed correlations, given that embryos from each patient are analysed as independent samples in most TLI studies [30].

Significant variations in early cleavage- stage morphokinetic variables according to the insemination technique were reported by a few TLI studies [26,57,58]. Lemmen et al. (2008) noted that ICSI derived four-cell embryos spent significantly shorter period as two-cell embryos compared to IVF-fertilised ones [57]. Comparing the early cleavage -stage morphokinetic variables (tPNf to t4) among IVF and ICSI- fertilised embryos, Bodri et al. (2015) observed a significant delay for IVF-fertilised embryos, on average between + 1.5 to +  1.1 hours [27]. Dal Canto et al. also reported a significant delay in early-cleavage timings (+1.4 and + 1.1 for t2 and t3 respectively) for IVF fertilised zygotes [58]. This average difference however, disappeared by the six- to eight-cell stage. IVF embryos developed slower than ICSI embryos, with significant differences in morphokinetic variables, ranging between + 1.2 and + 1.5 hours, both for early (t2 and t3) and late cleavage-stage parameters such as time to be five-cell (t5), seven-cells (t7) and nine-cell stages (t9) [26]. The average delay in early morphokinetic timings stems from the time needed for sperm penetration of the oocyte cumulus complex, interaction with the zona pellucida and finally the sperm-oocyte fusion, all of which are bypassed by ICSI procedure [59].

The objective of our study was to develop a TLI prediction model for live-birth probability based on Day 2 embryo transfers. We also investigated the effects of including and excluding female age in the model.

## Materials and methods

### Study population for building Day 2 transfer models

This retrospective study analyses data from 1,506 Assisted Reproductive Technology (ART) treatment cycles, all involving Day 2 embryo transfers. Among these, 865 cycles involved SET, and 641 involved double embryo transfers (DET). In total, 2,147 embryos were transferred. For cycles involving DET, the inclusion criteria required that either both embryos be either successfully implanted or neither, ensuring consistency in the data set.

Cycles with donor oocytes as well as cryopreserved embryos were not included in the study.

Data were collected from Klinikk Hausken in Haugesund, Norway, spanning from May 2011 to August 2018 and data for analysis were accessed in September 2018 first and then later in February 2020. All data were anonymised before the analyses were performed. Detailed stratification by age and live birth is not provided due to GDPR restrictions; only summary statistics and broad averages are reported.

### Ovarian stimulation, oocyte insemination, culture, and transfer

We utilised two ovarian stimulation methods: the GnRH agonist protocol and the ganirelix antagonist approach. Oocyte retrieval occurred 36 hours after administering hCG, guided by ultrasound. Sperm was collected either through ejaculation or via surgical techniques, the latter primarily for cases of male infertility, following WHO standards [60]. The fertilised zygotes were subsequently cultured in EmbryoSlide™ (Vitrolife, Sweden) within specific environmental conditions until prepared for transfer. Detailed descriptions of these procedures are provided in Sayed et al. (2022) [12].

### Time-lapse embryo monitoring and patient data

Time-lapse embryo assessments were performed daily using the EmbryoViewer software (Vitrolife, Denmark). The unique patient identifiers such as patient registration number and treatment cycle ID were entered in the EmbryoViewer based on information from the electronic medical journal, the IDEAS treatment database (Mellowood Medical, Canada). These included all patient characteristics (including BMI, female age, and infertility diagnosis) and treatment cycle data, as elaborated in Sayed et al. (2022) [10].

Time-lapse videos captured embryonic development, enabling monitoring and annotation of cell cleavage patterns, nucleation status, and cell cycle timings, all expressed as hours post-insemination (hpi). Both IVF and ICSI zygotes were annotated, and the insemination time noted. For ICSI cycles, the midpoint of the injection procedure was marked as the insemination time in the EmbryoScope. For IVF cycles, the moment the prepared semen sample was introduced to the oocyte-containing insemination dish was designated as the insemination time. Embryologists annotated cell cycle events and nucleation status in a sequential manner following a Standard Operating Procedure (SOP), reducing variability.

### Annotation of morphokinetics and nucleation status

Morphokinetic annotations, per Meseguer et al. (2011), were cell stage specific and included: fading of two pronuclei (tPNf), timings of embryo cleavages, and appearance of two (t2), three (t3), and four-cell stages (t4) [15]. Key annotations included second cell cycle duration (cc2 =  t3-t2). PN duration (VP) was also recorded for all insemination types. For Day 2 embryos, nucleation status was annotated as per Ciray et al. (2014) [18], with a focus on embryos with a single nucleus per blastomere [61]. Nucleation annotations at the two-cell stage and the four-cell stage have been elaborated in Sayed et al. (2022) [12].

### Embryo assessment and selection

On Day 2, embryos were chosen for transfer based on a morphological evaluation assessing blastomere size and symmetry, fragmentation degree, nucleation status and the absence of cleavage anomalies as described in Sayed et al. [12]. Nucleation errors and cleavage anomalies were utilised for excluding embryos from transfer and cryopreservation. Prior to the transfer of any embryo with impaired development, treating physicians were required to fully inform and obtain explicit consent from the couples. The number of embryos to be transferred followed the clinic’s standard operating procedure (SOP) as elaborated in Sayed et al. (2022) [12].

### Model development and statistical analysis

This study aimed to develop predictive models to accurately forecast clinical outcomes. To assess model performance, we primarily used the Area Under the Curve (AUC) derived from Receiver Operating Characteristic (ROC) curves. Higher AUC values indicate better predictive accuracy, showing how well the model distinguishes between different outcomes.

We also used the Akaike Information Criterion (AIC) to select the most relevant variables. Adding too many variables can complicate a model without enhancing its accuracy, therefore we ensured that additional variables did not increase the AIC. This approach helps balance simplicity and predictive power by explaining as much data variation as possible using only essential covariates.

#### Covariates and thresholds.

To build the models, we evaluated combinations of factors, such as the percentage of cell fragmentation, nucleation error status (binary: absent or present), the time interval between stages t2 and t3 (in hours), and the timing of t2 (in hours post-insemination, or hpi). The time between t2 and t3 was further categorised into short, medium, and long cell cycles, following the guidelines established by VerMilyea et al. (2014) [62].

In threshold analysis, a simple binary decision rule classified t4 values below a defined limit as 1 (positive result) and values above as 0 (negative result). For consistency, missing t4 values were set to 0.

#### Generalized additive mixed model (GAMM).

The central statistical model used was a Generalized Additive Mixed Model (GAMM), chosen for its flexibility in modelling non-linear relationships while accounting for patient-specific differences. For example, we explored how t2 (time to the 2-cell stage) might influence the outcome. Unlike many models that assume a linear effect, GAMMs can capture complex, non-linear patterns through smooth functions, providing a more accurate representation of this relationship.

The GAMM framework also allows to include ‘random effects’ to handle individual patient variability. Specifically, the random effect accommodates patient-specific variations, improving the model’s predictive power and generalisability. Hereby, we accounted for the potential effects of single versus double embryo transfers (SET/DET) in the model, which could impact outcome predictions.

#### Smoothing parameter estimation.

For smoothing parameter estimation, we chose the Restricted Maximum Likelihood (REML) method over Maximum Likelihood (ML). ML does not fully account for unpenalized and parametric effects when optimising smooth terms, potentially leading to less reliable estimates. REML integrates these effects into the likelihood calculation, providing solid estimates. This method aligns better with the data’s clinical nature, allowing to capture complex patterns without overfitting.

Complexities involved when using a GAMM are further elaborated in a Supporting information file (S1 file).

#### Model structure and covariates.

In the GAMM, the smooth terms capture the non-linear effect of the time to the 2-cell stage and age on KID_LB, while the categorical covariate adjusts for different cycle speed categories (short, medium, long) that may influence the outcome. By incorporating only the most relevant variables, the model balances interpretability with predictive strength, providing clinically meaningful insights. The supporting informing file (S2 File) outlines the comparison of GAMM models with other approaches.

#### Model validation.

We validated the models AUC using a bootstrap approach with 5-fold cross-validation. For each fold of the 5 folds, a GAMM was fitted to the training set. Within each fold, 200 bootstrap resamples were created, resulting in 1,000 AUC values overall. Clustering avoided the DET cycles to be split during the cross-validation. The mean AUC and its 95% confidence interval across all folds provided an overall measure of model performance.

#### Stratification analysis.

To examine the impact of age, we stratified data based on both age groups and model-generated probability scores. Age was divided into three equal segments (lower, middle, upper tiers), representing one-third of the age distribution. The probability scores calculated by the Age Model were similarly categorised into three stratification levels. Combining these two factors created nine subgroups, which allowed us to explore the model’s performance across different age and score strata.

All statistical analyses and validation were performed in R (R Foundation for Statistical Computing, Vienna, Austria).

### Ethical approval

This study protocol was approved by the Regional Committee for Medical and Health Research Ethics (REC) (2017/1610). The data analysis being retrospective did not consider the need for a specific consent form pertaining to the study. The consent form signed by the couple at the start of treatment did not specifically mention the study and hence based on REC recommendations, a new consent form was sent out to all couples who underwent ART treatment at the clinic during the study period. Couples who did not wish their data to be included in the study were requested to send written replies to the clinic. 26 couples who did not wish to participate in the study had their replies documented in their patient files in IDEAS and their embryo annotations and data removed from the analysis. This study protocol was approved by REC. All data pertaining to the study period were fully anonymised before accessing them for analysis.

## Results

The initial phase of the analysis involved assessing the individual predictive capabilities of various variables for model applicability, as summarised in Table 1. The optimal value for the t4-threshold was 40.5 hours, using a binary (0,1) approach.

**Table 1:** AUC and *P* values for single variables, using GAMM modelling.

Variable	AUC	*P*
**Fragmentation**	0.543	< 0.02
**Second cell cycle**	0.551	Long < 0.03. Short < 0.008
**Multinucleation**	0.559	< 0.0002
**t4-threshold**	0.609	< 3 ∙ 10^-9^a^
**t2**	0.622	< 2 ∙ 10^-16^a^
**Age**	0.735	< 2 ∙ 10^-16^a^

^a^Scientific notation. This is a method of expressing very large or small numbers through a coefficient multiplied by ten raised to a power.

### Construction of prediction models

The following variables were included in the models: status of multinucleation, the duration of the second cell cycle categorised as short, medium, and long, and the timing of the two-cell stage (t2). Fragmentation and the t4-threshold did not fulfil the inclusion criteria described in Materials and Methods.

The first model ‘Base Model’ deliberately excluded age as a variable, while the second model ‘Age Model’ incorporated age. The Base Model excluding age achieved an AUC of 0.654, while the inclusion of age significantly enhanced the AUC to 0.772. The AUC and P values are shown in Table 2.

**Table 2:** AUC and *P* values for the prediction models without and with inclusion of female age.

Model	AUC	*P* for age	*P* for t2	*P* for MN (^a^)	*P* for short cycle	*P* for long cycle
**Age excluded**	0.654	–	< 6 ∙ 10^-06^b^	< 0.002	0.045	0.035
**Age included**	0.772	< 2 ∙ 10^-16^b^	<0.0006	0.025	0.062	0.056

^a^Multinucleation.

^b^Scientific notation.

Table 3 summarises the key performance metrics derived from a 5-fold bootstrap cross-validation process, applied to predictive models, respectively both excluding and including maternal age as a covariate.

**Table 3:** Five-fold Bootstrapping performance metrics of predictive models in ART cycles.

Model	AUC	95% CI for AUC
Base Model	0.641	0.621 - 0.672
Age Model	0.745	0.669 - 0.786

The AUC values decreased after bootstrapping compared to the initial results shown in Table 2. This reduction reflects the inherent variability captured by the bootstrapping process, which adjusts for overfitting from the initial model development phase. The fivefold cross-validation performed 200 times with unique random splits provided an assessment of model performance. The distribution of the 1,000 AUC values, for each of the two models, can be seen in supporting information figures S1 Fig and S2 Fig.

To evaluate the influence of maternal age, we stratified the data into three equal age groups. Similarly, the predicted probabilities generated by the age-inclusive model (Age Model) were divided into three equal intervals. These stratifications facilitated a direct comparison between predicted and observed live birth (LB) outcomes.

We segmented the age groups into three equal parts, and similarly, the age-inclusive model’s probability scores were divided into three equal levels. Within these stratifications, Table 4 displays the actual Live-birth (LB) ratios, while Table 5 presents the predicted LB ratios from the Age Model.

**Table 4:** Actual Live Birth (LB) ratios. The embryos are stratified by both age and model scores, providing 9 subsets.

	Lowest score	Medium score	Highest score
**Lowest age**	14.9%	31.7%	37.7%
**Medium age**	7.6%	16.7%	20.8%
**Highest age**	2.6%	6.0%	6.9%

**Table 5:** Live Birth (LB) ratio predictions from the Age Model. This table follows the stratification of Table 4.

	Lowest score	Medium score	Highest score
**Lowest age**	15.9%	29.5%	38.4%
**Medium age**	8.2%	16.7%	22.2%
**Highest age**	2.4%	4.8%	6.9%

When comparing Table 4 with Table 5, the correspondence between the actual and projected LB ratios can be assessed.

Table 6 shows the number of embryos in each of the 9 stratifications.

**Table 6:** Numbers of embryos. This table follows the stratification of Table 4.

	Lowest score	Medium score	Highest score
**Lowest age**	195	221	300
**Medium age**	249	227	240
**Highest age**	272	268	175

The average Live Birth ratios for the nine stratifications in Table 4 and Table 5 are very similar. There is a considerable Live Birth ratio span from the lowest age and highest score to the highest age and lowest score.

As a control for equal distribution, the sums for both 3 rows and 3 columns in Table 6 were calculated, ranging between 715 and 716.

Table 7 presents stratified AUC values from the Age Model, Table 8 shows AUC values from the Base Model, and Table 9 features AUC values from the Age Model, but with the age set to the average within three age stratifications.

**Table 7:** Stratified AUC values from the Age Model including age.

	Lowest score	Medium score	Highest score
**Lowest age**	0.723	0.545	0.588
**Medium age**	0.704	0.631	0.631
**Highest age**	0.785	0.721	0.697

**Table 8:** AUC values from Base Model excluding age.

	Lowest score	Medium score	Highest score
**Lowest age**	0.700	0.509	0.534
**Medium age**	0.625	0.523	0.546
**Highest age**	0.762	0.541	0.516

**Table 9:** AUC values from the Age Model but setting age to average for the three respective age groups.

	Lowest score	Medium score	Highest score
**Lowest age**	0.695	0.522	0.534
**Medium age**	0.630	0.515	0.547
**Highest age**	0.749	0.508	0.515

Table 7 to 9 follow the stratification from Table 4.

Table 8 shows stratified AUC values from the Base Model.

When comparing Table 8 with Table 9, the AUC values in the 9 stratifications are very similar.

### Validation

Regarding the delay in tPNf for IVF compared to ICSI, we observed a 0.1-hour difference, less than the timings reported in previous studies [26,27,58,63].

**Fig 1:**
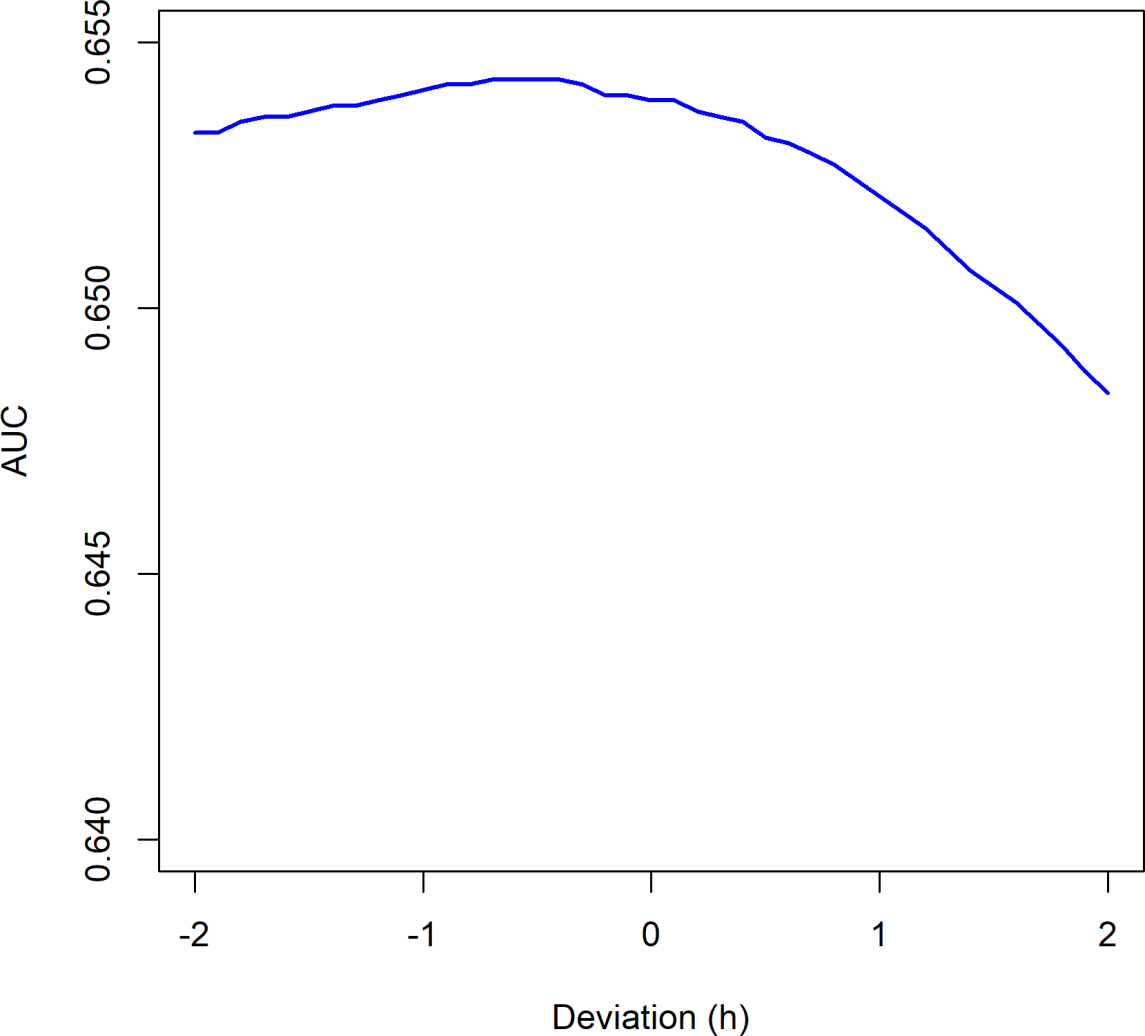
ICSI t2 timing manipulation± 2 hours, Base Model. Optimal AUC at -0.5 hours.

**Fig 2:**
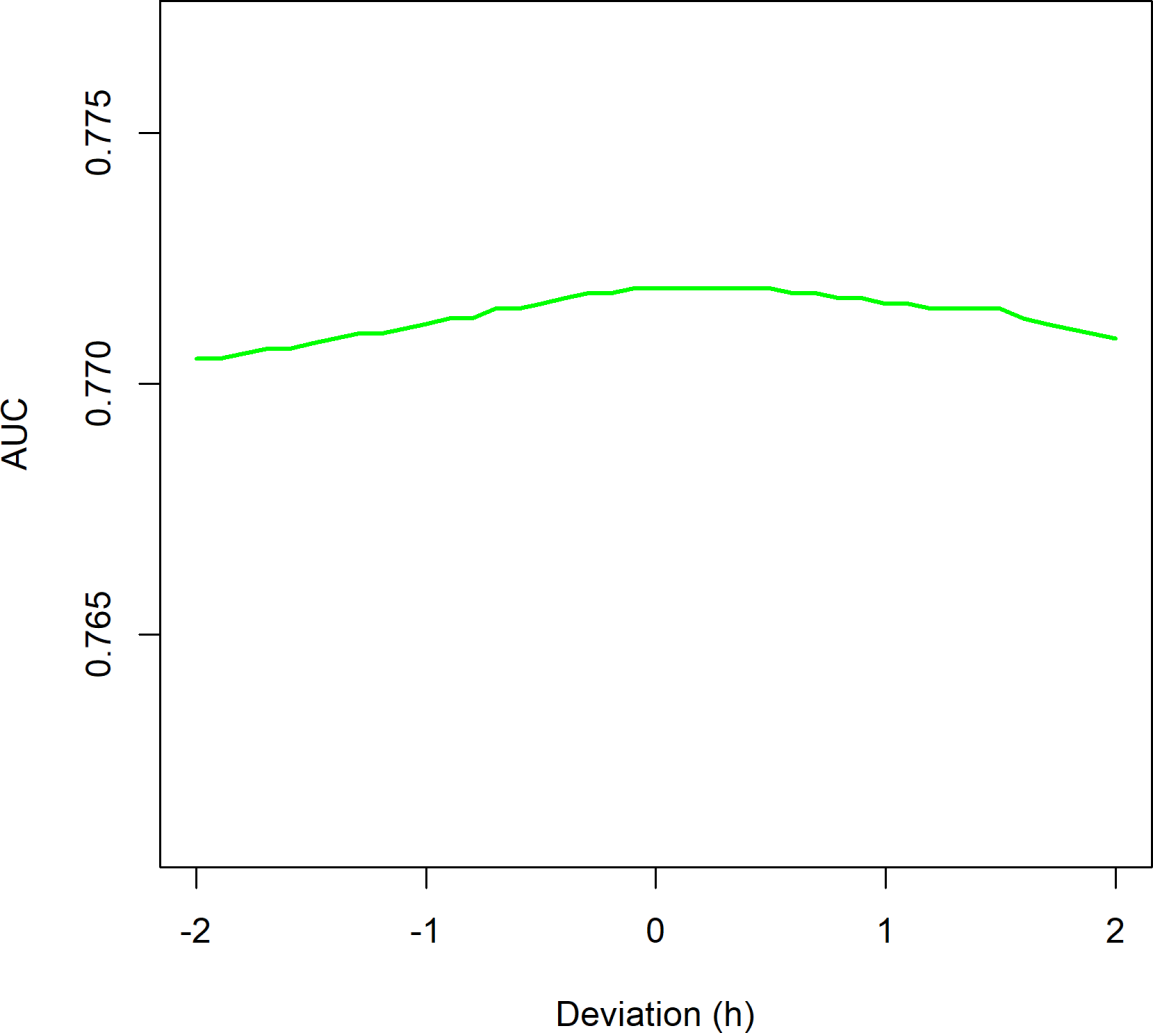
ICSI t2 timing manipulation± 2 hours, Age Model. Optimal AUC at + 0.3 hours.

Fertilisation timing varies between IVF and ICSI, leading to differences in the timing of morphokinetic events, with IVF on average experiencing a delay in fertilisation compared to ICSI. The potential implication of this delay is investigated in Fig 1 and 2, where the sensitivity of predictive models to alterations in the timing of the two-cell stage (t2) in ICSI embryos is explored. For this purpose, t2 timings for ICSI embryos were artificially adjusted within a range of -2 to + 2 hours.

Fig 1 illustrates that, in the Base Model, the optimal adjustment for maximising AUC is an addition of 0.5 hours for t2. This adjustment minimises the AUC difference (0.0055) from the original timing (AUC 0.654) to the lowest AUC observed at a ±  2-hour displacement.

Fig 2 demonstrates that, in the Age Model, a slight delay of 0.3 hours in t2 timing optimises the AUC. The difference between the AUC for non-adjusted timings (0.772) and the lowest AUC at a ±  2-hour displacement is smaller (0.001).

The bootstrap AUC values could not be provided here, because these very small differences seen here are not suited for a process that has an element of noise.

## Discussion

In this retrospective study, we developed and compared two time-lapse imaging (TLI) prediction models to assess live birth probabilities following Day 2 embryo transfers. Specifically, we examined the effects of including and excluding maternal age as a covariate in the models. An examination of the AUC values presented in Tables 7 and 8 is important to understand the nuances of integrating age as a variable in predictive modelling.

Table 9 presents the AUC values for the model integrating age as a variable (Age Model), with age averaged within predefined stratifications. This contrasts with the models in Tables 7 (Age Model using actual patient ages) and Table 8 (Base Model, excluding age).

Interestingly, Table 9 shows that averaging age within stratifications results in AUC values similar to the Base Model, suggesting that averaging age does not improve predictive accuracy within an embryo cohort. This leads to a complex interpretation.

The Age Model does have a much higher predictive ability that the Base Model. So, this model can be suited for counselling to provide a probability of success.

But when the embryos are to be evaluated, the Base Model predictions will be a better choice than the Age Model, as this model has a comparable predictive ability and further, due to the strong correlation between age and IVF outcomes, incorporating age into the model might even deteriorate the reliability of the predictions.

In practical terms, including age might slightly adjust model calculations, potentially improving accuracy for certain patient groups. However, the lack of significant additive effects from age as a predictor indicates minimal enhancement in clinical utility. This supports the premise that while age may add context for broader prognostic discussions, its inclusion does not offer added value in decision-making within homogeneous treatment cohorts.

Our results partially contradict van Marion et al. (2023), who reported improvements in predictive performance when including age in their models [64]. However, our analysis highlights that while age can provide additional information in broader contexts, its impact is questionable within single treatment cohorts. This distinction underscores the need for context-specific evaluations of predictive models.

Milewski et al. (2017) [65] also incorporated age in their TLI model, reporting an AUC of 0.75, closely aligning with our Age Model results. Conversely, van Marion et al. (2023) reported lower AUC values of 0.65 and 0.60 in their two models, further emphasising variability in the effectiveness of age as a predictor [64].

Only few TLI models address Day 2 transfers. Desch et al. (2017) [13], for example, used Generalised Estimating Equations (GEE), which differ from the Generalised Additive Mixed Models (GAMMs) applied in our study. Their multivariate analysis included maternal age, time of two-cell formation, multinucleation at the two- and four-cell stages, partially overlapping with our inputs. Similarly, the EEVA model (VerMilyea et al., 2014) [62], while not specific to Day 2, includes variables within the same timeframe.

The observed differences in AUC values between Table 2 and Table 3 highlight the value of using bootstrapped cross-validation in mitigating overfitting and evaluating model performance across varied datasets. The differences in AUC values reflect the importance of robust validation methods to ensure model reliability.

We explored the use of alternative performance metrics beyond the AUC to evaluate model performance in a broader context. Specifically, we considered commonly used metrics such as the F1 and F2 scores, sensitivity, and specificity. However, due to the observed average success ratio of 1:4 none of these metrics provided reliable or meaningful results.

Furthermore, the use of a standard confusion matrix was meaningless, as no predicted probabilities in our model exceeded 0.5.

A potential limitation of the models relates to the uncertainty surrounding fertilisation timing, which affects the precision of t2 (time to two-cell) measurements. In ICSI cycles, insemination timing was recorded as the midpoint of the injection procedure, while in IVF cycles, it was less precise. Adjusting morphokinetic parameters to pronuclear fading (tPNf), as suggested by Bodri et al. [27] and Dal Canto et al. [58], could reduce these discrepancies and remove inconsistencies in time to reach early cleavage events [66,67]. Despite these challenges, t2 remains a consistent and reliable predictor of success, aligning with findings that early cleavage correlates with higher success rates [68,69].

Importantly, our analysis demonstrated that adjustments to t2 timings (±2 hours) have negligible influence on predictive performance (Fig 1 and 2). This reinforces t2’s robustness as a variable, even amidst fertilisation timing uncertainties, and argues against excluding t2 due to perceived imprecision. Instead, excluding t2, due to the perceived uncertainty, could result in poorer predictions.

The predictive models for the likelihood of live births following IVF treatment were developed based on a retrospective analysis of time-lapse data from Day 2 embryo transfers performed at the clinic. Day 2 transfers were the preferred norm during the study period and the decision to schedule transfers was mainly based on the clinician’s availability and preference, as well as flexibility in scheduling transfers for the convenience of patients travelling from afar for treatment. Applying our findings to blastocyst selection and Day 5 transfer might be beneficial for the patient with regards to live birth.

Bias introduced by using models designed for general embryo selection without accounting for transfer day remains a concern. For instance, Lassen et al. (2023) [70] demonstrated higher predictive performance using separate AI models for cleavage-stage and blastocyst transfers compared to a combined model. This indicated that embryo transfer day must be considered in model development.

While addressing potential confounders, we observed higher AUC values with increasing age [71,72], consistent with the known negative correlation between age and clinical outcomes. However, in embryo selection models, including age may increase AUC values without improving ranking performance within an individual’s embryo cohort [70]. This aligns with our findings that age, while informative for broader prognostic contexts, does not enhance predictive efficacy within single treatment cohorts.

Thus, an age-exclusive model can be presumed to be better suited for embryo selection processes, whereas an age-inclusive model could guide patient-specific treatment strategies.

## Conclusion

In this study, we developed two predictive models: one excluding age (AUC value of 0.641), and another incorporating age (AUC value of 0.745, bootstrap derived). Despite the significant difference in AUC values, practical predictive performance for embryo selection in a cohort is better performed using a model that excludes age. Conversely, a model utilising age data will have clearly better predictions for general counselling, e.g., before treatment onset.

Over-reliance on age-inclusive models due to higher AUCs, without critically assessing clinical relevance, risks misguiding decision-making.

Clinics may favour age-inclusive models for their higher performance metrics. However, this could obscure the effectiveness of age-exclusive models, especially in homogeneous patient cohorts. So, within the complexity of the models using or not using age data in this study, there is a clear risk of making suboptimal decisions based on perceived model performance. In some cases, the actual performance may be clearly lower than expected.

This study thus underscores the importance of assessing actual performance and clinical relevance over purely statistical metrics.

The fundamental deductions from this study, comparing models with and without age are independent of embryo transfer day (Day 2, Day 3, or blastocyst stage).

Further independent validation is required before the present models can be reliably integrated into clinical decision-making.

## Supporting information

S1 File Overview of GAMM functions and parameter tuning.
(DOCX)

S2 File Strengths of GAMMs compared to other modelling approaches.
(DOCX)

S1 Fig Bootstrap histogram not using Age.
(TIFF)

S2 Fig Bootstrap histogram using Age.
(TIFF)

S3 Fig Odds ratio t2 spline not using Age.
(TIFF)

S4 Fig Odds ratio t2 spline using Age.
(TIFF)

S5 Fig Odds ratio age spline not using Age.
(TIFF)
