# Supplementary material for: Predicting IVF live -birth probability using time-lapse data: Implications of including or excluding age in a day 2 embryo transfer model
Source: PLoS One. 2025 Feb 25;20(2):e0318480. doi: 10.1371/journal.pone.0318480 (PMC11856505; doi:10.1371/journal.pone.0318480)
Supplement: S1 File — Overview of GAMM functions and parameter tuning. (DOCX) [file pone.0318480.s001.docx]

**Supporting Information**

**Overview of GAMM functions and parameter tuning**

This appendix provides a brief overview of the key components of the Generalized Additive Mixed Models (GAMMs) used in this study, focusing on smooth terms, model flexibility, and parameter tuning for basis dimension (k) and smoothing penalty (gamma). The appendix also outlines how the EDF reflects smooth term complexity and describes the process used to balance flexibility, interpretability, and performance.

**Basis Dimension (k)**

The "basis dimension" (k) is an important concept in both Generalized Additive Models (GAMs) and Generalized Additive Mixed Models (GAMMs). It defines the maximum number of "building blocks", called basis functions, that the model can use to create smooth curves when fitting data. In simple terms, it controls how flexible the curve can be, whether it follows broad trends or captures smaller details in the data.

In this study, we used a type of basis function called "thin plate regression splines" to create the smooth curves. Thin plate splines are often used because they are both flexible and efficient at capturing non-linear patterns

Thin plate splines do not require the user to place "knots" manually, which are points where the curve is allowed to bend more. Instead, thin plate splines automatically create a smooth fit across the entire range of data by using what is called a "low-rank basis." This means the model uses fewer building blocks to create the curve, which both keeps the computations efficient and prevents the model from becoming overly complex.

Higher values of k allow the model to capture more complex patterns in the data, but excessive flexibility can lead to overfitting. Therefore, careful selection of k is crucial to balance model complexity and interpretability.

**Effective Degrees of Freedom (EDF)**

The EDF quantifies the flexibility of a smooth term in a GAMM. EDF measures how much "flexibility" the model applies to fit a specific smooth term.

If the EDF approaches the value of k, it suggests that the basis dimension may be limiting, as the smooth term is using the maximum flexibility allowed. In such cases, increasing k may be necessary to capture more complex patterns, depending on the context.

Conversely, if the EDF is substantially lower than k, it suggests that additional flexibility is unnecessary and that the smooth term is sufficiently constrained.

A low EDF indicates a smoother, more linear relationship, while a higher EDF indicates greater complexity or "wiggliness." Monitoring EDF values helps ensure an appropriate balance between model complexity and interpretability.

**Smoothing Penalty (Gamma)**

The parameter "gamma" scales the penalty applied to the smooth terms relative to the likelihood, thereby controlling the trade-off between model fit and overfitting. In practical terms, gamma adjusts the penalty applied to each basis function, indirectly influencing how closely the smooth terms fit the observed data.

**Grid Search for Parameter Tuning**

A grid search was conducted over the following parameter ranges to optimise the model:

“k values: 10, 20, 30, 40 (default value is 20).”

“gamma values: 0.5, 1, 1.75, 2.5 (default value is 1).”

The model performance was assessed using AUC.

The optimisation process was performed by running the model on the entire dataset for each of the 16 above initial iterations.

In subsequent iterations to maximise AUC, the search space was refined until the final values of k and gamma were determined to two decimal places, ensuring precision in parameter estimation without placing excessive demands on CPU resources.

**Parameter Stability and AUC Differences**

Among the initial 16 tested combinations of k and gamma, the difference between the lowest and highest AUC values for the model excluding age was 0.189%. The difference between the lowest AUC value and the optimised AUC value was 0.197%.

For the model including age, both differences were 0.327%.

These results indicate that, for the present modelling, the "landscape" of AUC values across the parameter space is quite “flat”. Other data inputs to GAM/GAMM models may experience more "bumpy" landscapes during parameter optimisation.

**Results and Insights**

**EDF Values and Model Fit**

In both models, the EDF values for the smooth terms did not approach the upper bound of k, indicating that the chosen basis dimension (k) was sufficient to capture the relationships without imposing unnecessary constraints or requiring additional flexibility.

**Smoothing Penalty (Gamma)**

The optimal gamma values for both models balanced flexibility to smoothness, ensuring that the models avoided overfitting while adequately capturing the underlying relationships in the data.

**Odds Ratios and Smooth Terms**

The odds ratio, derived from the smooth terms in the GAMM, quantifies the change in the odds of the binary outcome (KID_LB) associated with a one-unit increase in the predictor, while holding other predictors constant. For this study, two smooth terms, t2 (representing temporal effects) and Age, were analysed.

Supporting figures S3 Fig, S4 Fig, and S5 Fig all exhibit smooth, non-linear curves without signs of excessive fluctuation, indicating well-optimised model settings.
