# Supplementary material for: Predicting IVF live -birth probability using time-lapse data: Implications of including or excluding age in a day 2 embryo transfer model
Source: PLoS One. 2025 Feb 25;20(2):e0318480. doi: 10.1371/journal.pone.0318480 (PMC11856505; doi:10.1371/journal.pone.0318480)
Supplement: S2 File — Strengths of GAMMs compared to other modelling approaches. (DOCX) [file pone.0318480.s002.docx]

**Supporting Information**

**Strengths of GAMMs compared to other modelling approaches**

This Supplemental text briefly compares GAMMs with other commonly used modelling approaches. It is important to note that such comparisons may be influenced by subjective interpretations and context-specific factors.

**Linear and Logistic Regression models**

Strengths: Simple, interpretable, and computationally efficient.

Limitations: These models assume linear or log-linear associations between predictors and outcomes. However, biological processes, such as embryo development and implantation, often do not follow such simple trends. If relationships between variables and outcomes are non-linear, as observed here, linear models may underfit the data.

**Decision Trees and Random Forests**

Strengths: Can handle non-linear relationships and interactions without the need for manual feature engineering.

Limitations: Decision trees tend to overfit unless carefully pruned, while random forests, though more robust, in our view lack the smooth, interpretable relationships offered by GAMMs. Instead, random forests produce stepwise predictions, which may obscure trends and make it difficult to infer smooth changes in probabilities over continuous predictors like age.

While useful for predictive tasks, decision trees and random forests were less suitable for our purpose due to their lack of interpretability for continuous relationships.

**Deep Learning Models**

Strengths: Flexible and capable of modelling complex interactions and non-linearities.

Limitations: Deep Learning Models require large datasets to achieve optimal performance. Deep Learning can hence tend to overfit on smaller datasets, as our present dataset. Further , they largely function as "black boxes," making it challenging to understand how input variables influence the output.

Deep Learning Models were not considered in our study due to the cumbersome setup, low interpretability, and the moderate size of our dataset.

**Generalized Linear Mixed Models (GLMMs)**

Strengths: Utilise random effects and handle hierarchical structures in data.

Limitations: GLMMs assume a predefined functional form for relationships between variables, which limits their ability to model complex, non-linear associations.

Although GLMMs can handle random effects, they were not suitable for this study due to their linearity assumptions, excluding non-linear functions for continuous predictors.

**Generalized Estimating Equations (GEE)**

Strengths: GEEs handle correlated data and repeated measures by modelling the average effect across the population rather than individual-specific effects. They offer flexible working correlation structures to account for within-subject dependencies, making them well-suited for longitudinal and clustered data.

Unlike GLMMs, GEEs do not include subject-specific random effects and therefore cannot capture individual-level variability.

While GEEs are robust for analysing correlated data and provide interpretable, population-averaged effects, they were less suitable for this study due to the need to model both non-linear associations and individual-specific variability.

**Key advantages of GAMMs for this study**

GAMMs use smooth functions to capture non-linear associations between predictors and outcomes, which is particularly appropriate for biological processes like live birth prediction, where simple linear trends are unlikely to apply.

GAMMs provide visual representations of predictor effects, showing smooth curves that make the relationships easier to interpret and explain.

GAMMs include random effects to account for correlated observations, such as multiple embryos from the same patient. This avoids pseudoreplication and ensures that within-patient variability is properly accounted for.

Penalised smoothers prevent the model from fitting noise by controlling the 'wiggliness' of smooth terms. This balance between flexibility and parsimony is crucial for robust performance, particularly with moderate dataset sizes.

**Conclusion:**

In summary, GAMMs were chosen because they balance flexibility, interpretability, and robustness. GAMMs offer the ability to model complex, non-linear patterns while remaining accessible and meaningful for clinical decision-making. This makes them well suited to predicting live birth outcomes in the context of IVF treatment.
