# Supplementary figures and images for: Predicting IVF live -birth probability using time-lapse data: Implications of including or excluding age in a day 2 embryo transfer model
Source: PLoS One. 2025 Feb 25;20(2):e0318480. doi: 10.1371/journal.pone.0318480 (PMC11856505; doi:10.1371/journal.pone.0318480)

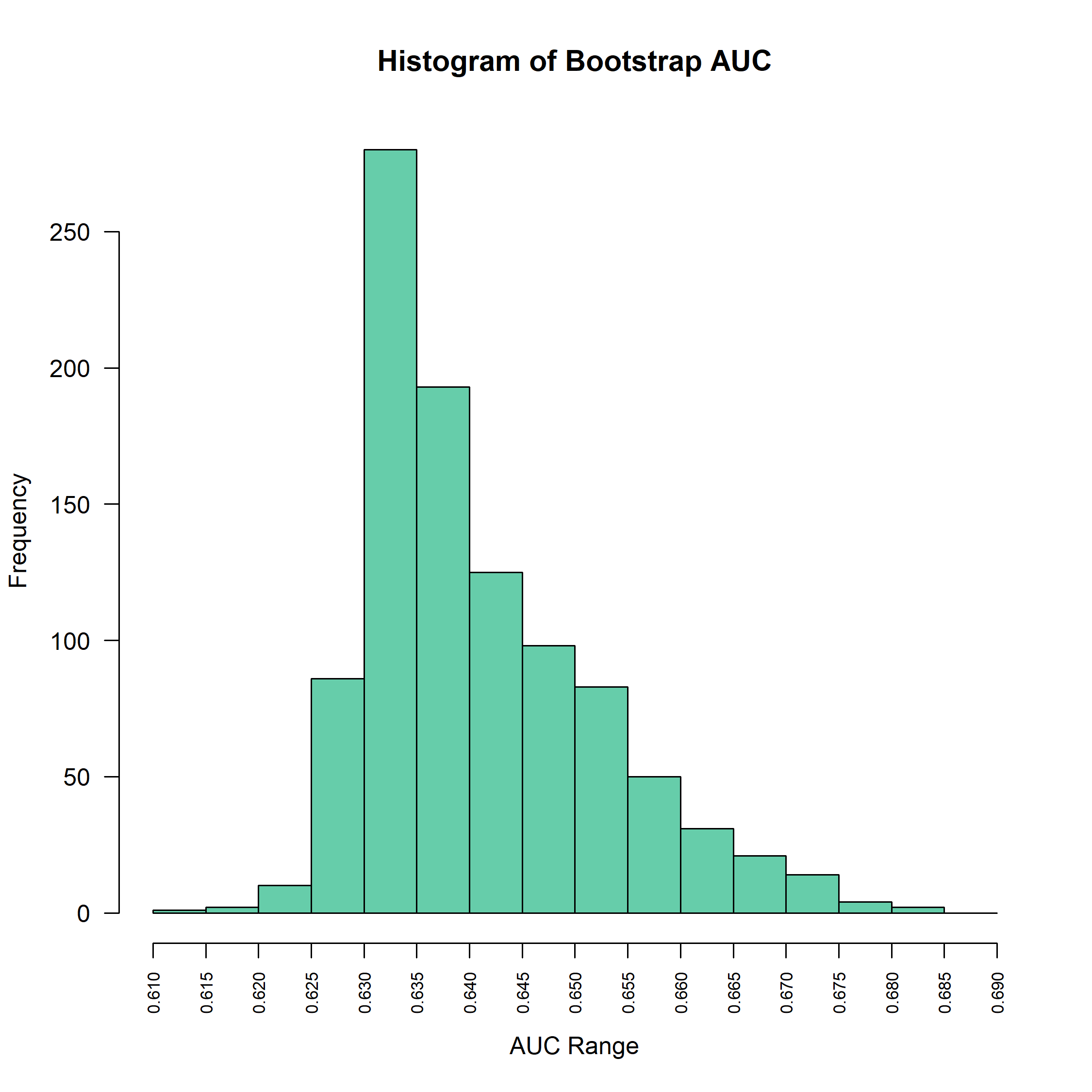

Supplement: S1 Fig — Bootstrap histogram not using Age. (TIFF) [file pone.0318480.s003.tiff]

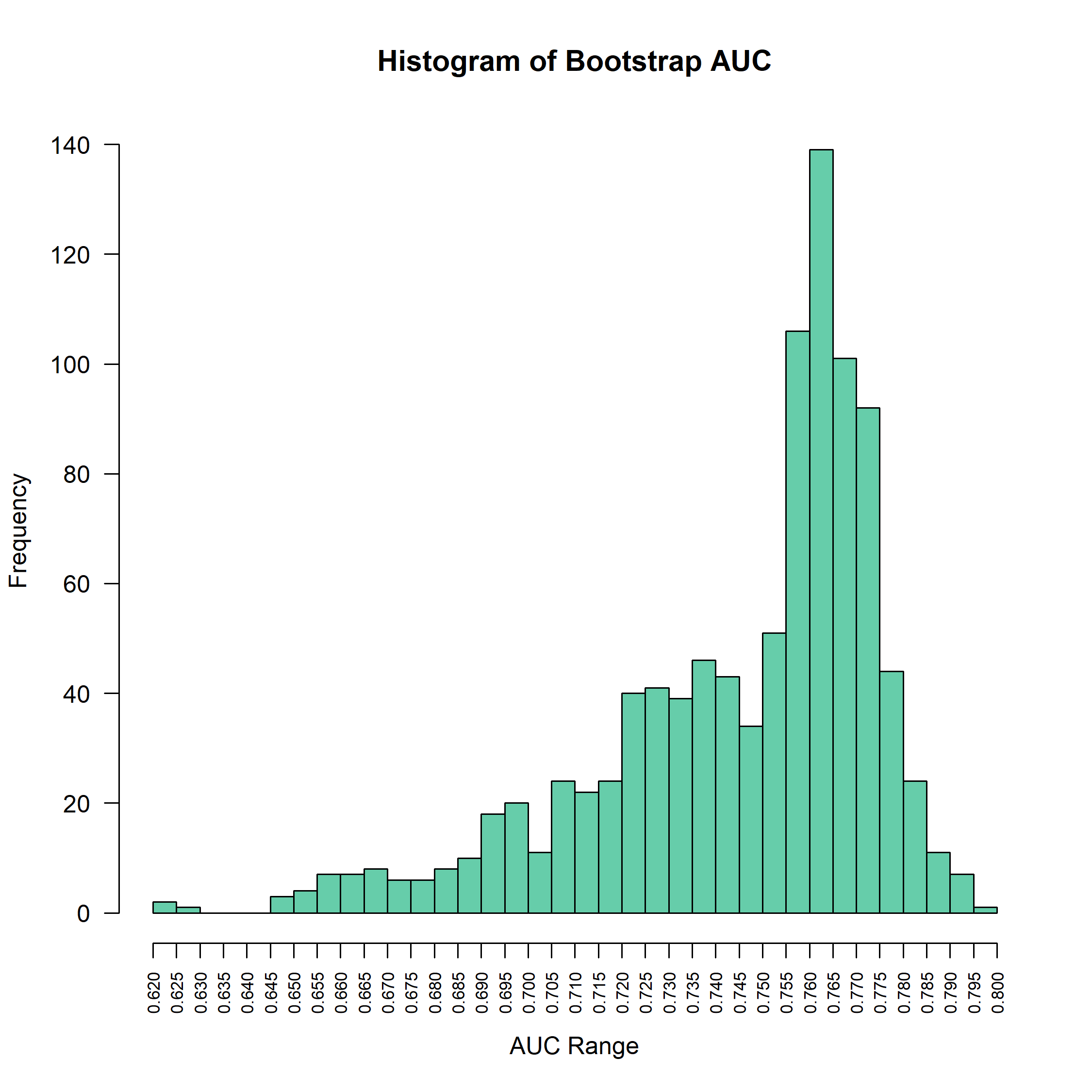

Supplement: S2 Fig — Bootstrap histogram using Age. (TIFF) [file pone.0318480.s004.tiff]

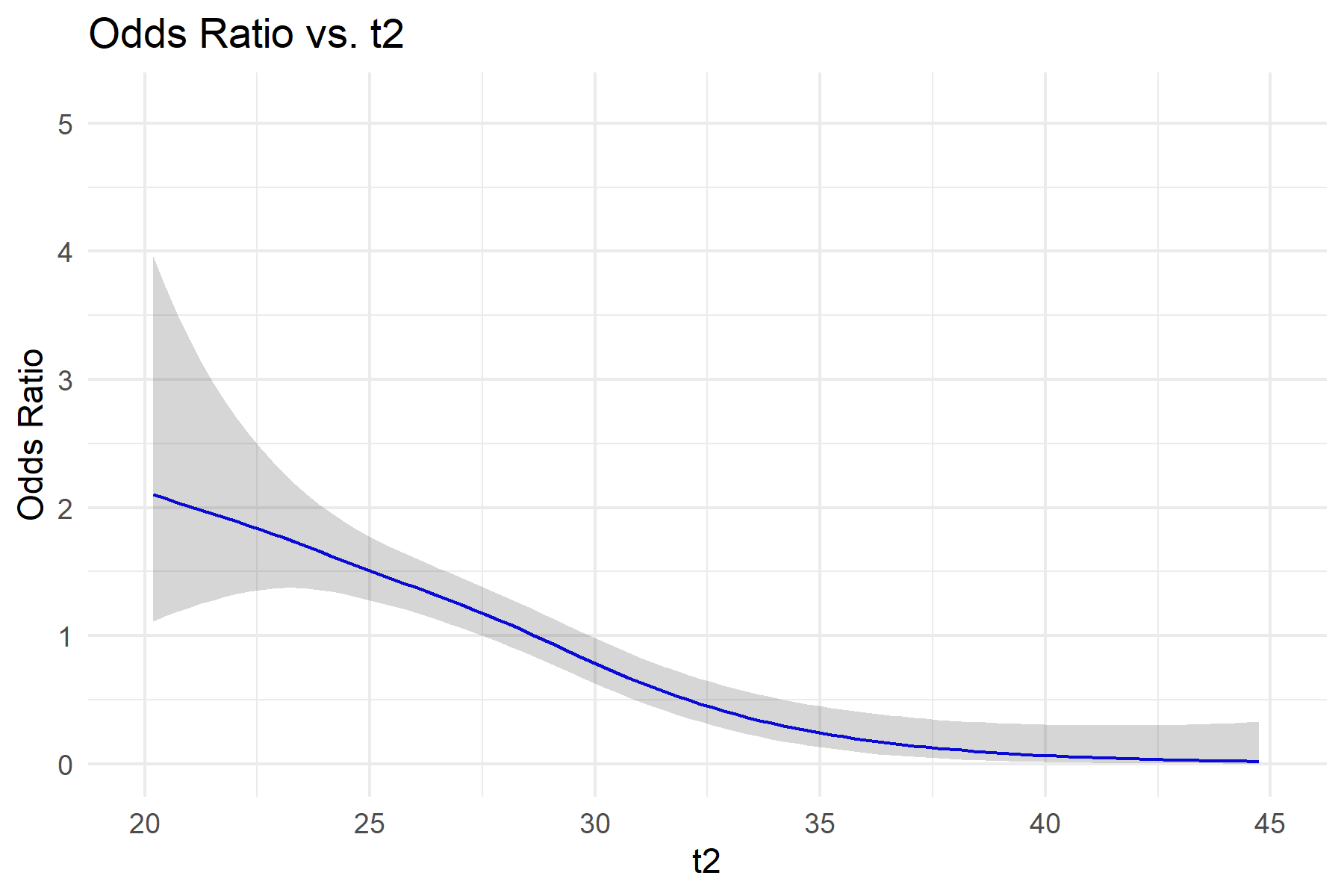

Supplement: S3 Fig — Odds ratio t2 spline not using Age. (TIFF) [file pone.0318480.s005.tiff]

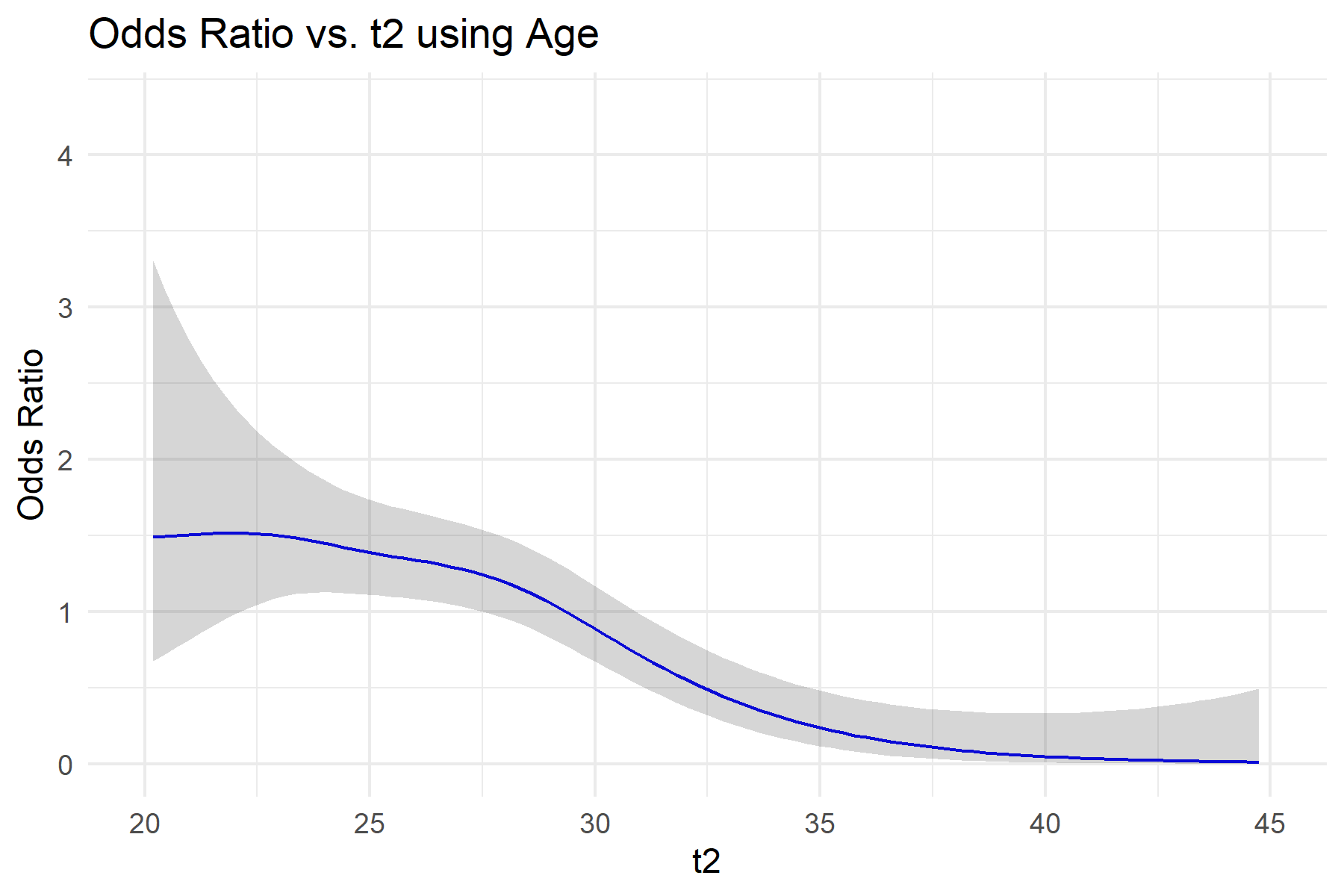

Supplement: S4 Fig — Odds ratio t2 spline using Age. (TIFF) [file pone.0318480.s006.tiff]

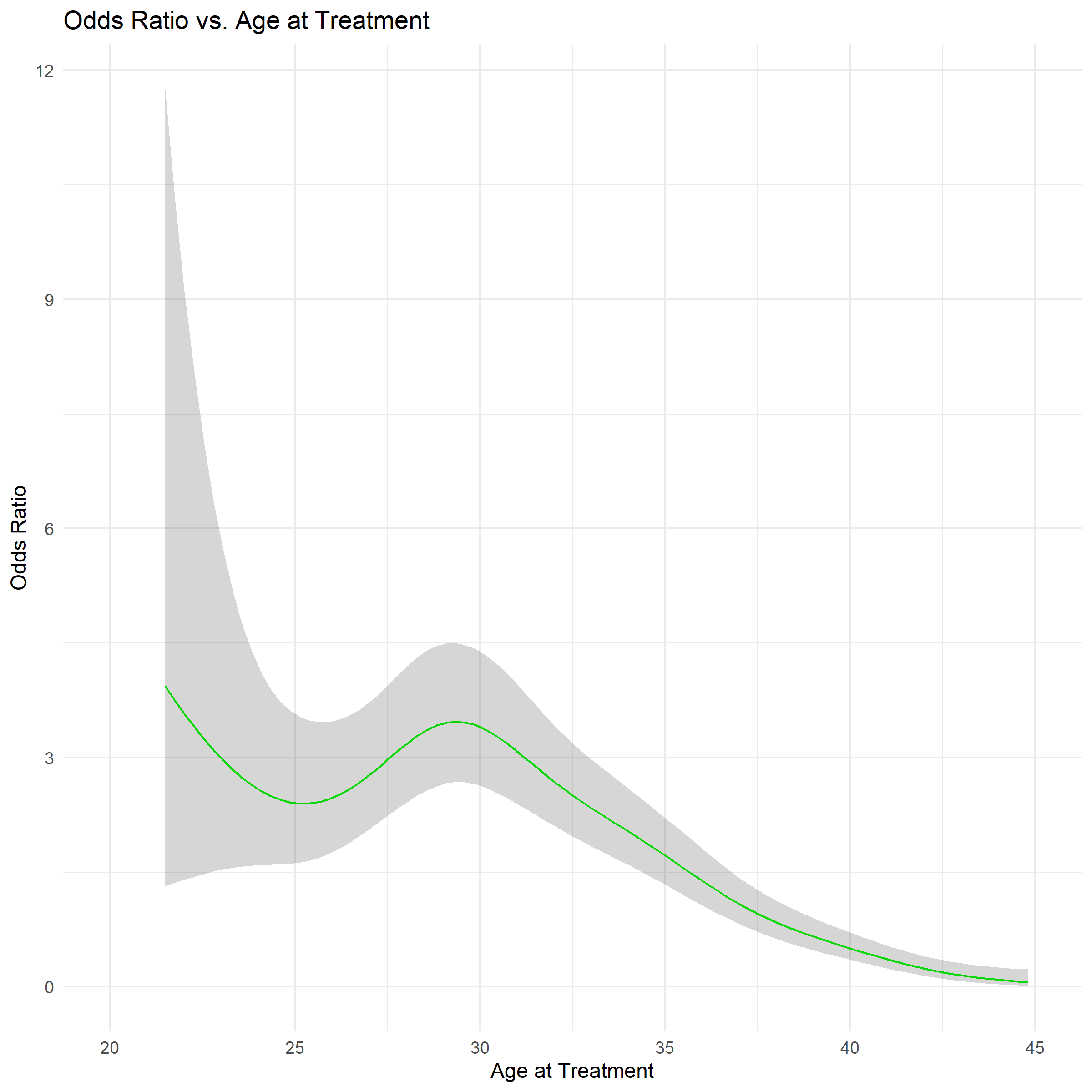

Supplement: S5 Fig — Odds ratio age spline not using Age. (TIFF) [file pone.0318480.s007.tiff]
